# Methanol extract of *Nigella sativa* seed induces changes in the levels of neurotransmitter amino acids in male rat brain regions 

**DOI:** 10.1080/13880209.2017.1302485

**Published:** 2017-03-21

**Authors:** Tarek El-Naggar, María Emilia Carretero, Carmen Arce, María Pilar Gómez-Serranillos

**Affiliations:** a Department of Pharmacology, Faculty of Pharmacy, Universidad Complutense, Madrid, Spain;; b Chemistry of Natural and Microbial Products Department, National Research Center, Cairo, Egypt;; c Department of Biochemistry, Faculty of Pharmacy, Universidad Complutense, Madrid, Spain

**Keywords:** Ethnopharmacology, aspartate, glutamate, glycine, γ-aminobutyric acid, HPLC

## Abstract

**Context:***Nigella sativa* L. (Ranunculaceae) (NS) has been used for medicinal and culinary purposes. Different parts of the plant are used to treat many disorders.

**Objective:** This study investigates the effects of NS methanol extract on brain neurotransmitter amino acid levels.

**Materials and methods:** We measured the changes in aspartate, glutamate, glycine and γ-aminobutyric acid in five brain regions of male Wistar rats after methanol extract treatment. Animals were injected intraperitoneally with saline solution (controls) or NS methanol extract (equivalent of 2.5** **g/kg body weight) and sacrificed 1 h later or after administering 1 daily dose for 8 days. The neurotransmitters were measured in the hypothalamus, cortex, striatum, hippocampus and thalamus by HPLC.

**Results and discussion:** Results showed significant changes in amino acids compared to basal values. Glutamate increased significantly (16–36%) in the regions analyzed except the striatum. Aspartate in the hypothalamus (50 and 76%) and glycine in hippocampus (32 and 25%), thalamus (66 and 29%) and striatum (75 and 48%) also increased with the two treatment intervals. γ-Aminobutyric acid significantly increased in the hippocampus (38 and 32%) and thalamus (22 and 40%) but decreased in the cortex and hypothalamus although in striatum only after eight days of treatment (24%).

**Conclusion:** Our results suggest that injected methanol extract modifies amino acid levels in the rat brain regions. These results could be of interest since some neurodegenerative diseases are related to amino acid level imbalances in the central nervous system, suggesting the prospect for therapeutic use of NS against these disorders.

## Introduction

*Nigella sativa* L. (Ranunculaceae) (NS) is a dicotyledon that commonly grows in the Middle East, Eastern Europe and Eastern and Central Asia. NS seeds and oil have been used for thousands of years as a spice and food preservative, as well as in natural remedies (Salem 2005; Ahmad et al. 2013). The herb has been regarded as a valuable remedy in hepatic and digestive disorders. The seeds have traditionally been used for their diuretic, diaphoretic, stomachic and digestive properties, and as a liver tonic; as a mixture with other ingredients, they are used against diarrhea, indigestion, dyspepsia and belching; they also improve foul breath in the mouth. At present, NS seeds are given with buttermilk to cure persistent hiccups and are also useful for loss of appetite, vomiting, dropsy and puerperal diseases. They are used as an emmenagogue in obesity and dyspnea. They have antibilious properties and are administered in intermittent fevers (Nadkarni 1976; Ramadan 2007; Paarakh 2010). Inhalation of fried seeds relieves cold and catarrh. They have also been used for chronic headache and migraine (Usmanghani & Alam 1997; Ramadan 2007). A decoction of the seeds with sweet oil creates a useful treatment for skin diseases. They have been useful against mercury poisoning, sores and leprosy (Evans 2009). After grinding the seeds and soaking in water, the resulting substance improves swelling in the hands and feet. NS is also used topically against leucoderma, alopecia, eczema, freckles and pimples (Usmanghani et al. 1997). NS seeds have also been used for their anthelmintic and antibacterial properties (Kapoor 1990; Bakathir & Abbas 2011).

Many active ingredients have been isolated in NS, including thymoquinone, thymohydroquinone, dithymoquinone, thymol, carvacrol, nigellicine and α-hederin (Khan 1999; Morikawa et al. 2004a, 2004b; Ali & Blunden 2003; Ahmad et al. 2013; Akram Khan & Afzal 2016). Different pharmacological effects have been identified for NS, such as immune stimulation (Salem 2005), anti-inflammatory properties (Chehl et al. 2009; Alemi et al. 2013), anticancer properties (Khan et al. 2011; Randhawa & Alghamdi 2011), antimicrobial activity (Morsi 2000; Bakathir & Abbas 2011) and antirheumatoid arthritis activity (Gheita & Kenawy 2012). NS seeds and oil have also been reported to have calcium antagonist activity associated with cellular protection (Aqel 1992; Gilani et al. 2001; Khan et al. 2016). Moreover, the fixed oil extract of NS has greater antioxidant activity levels than those of thymoquinone, the most active ingredient in the volatile oil of this plant (Houghton et al. 1995; Burits & Bucar 2000; Kanter et al. 2005; Al-Naqeeb et al. 2009). Recent studies confirm that the antioxidant, free radical scavenging, and anti-inflammatory properties of NS (Akhtar et al. 2013) and its anticonvulsant effects are probably the result of an increase in GABAergic tone (Hosseinzadeh & Parvardeh 2004; Hosseinzadeh et al. 2005; Ilhan et al. 2005; Noor et al. 2012).

Our goal has been to achieve a better understanding of the action of methanol extract. Recently, the neuroprotective effect of NS on antiepileptic activity has been studied by direct oral administration of the extract suspended in saline solution in rats (Arafa et al. 2013). Similarly, though many studies have been completed on crude NS extract and its constituents, few have been performed on the extract available in its aqueous suspension dosage form (Akhtar & Riffat 1991; Chakravarty 1993; Akhtar et al. 2013) or methanol extract (Morikawa et al. 2004a, 2004b). In previous studies, we demonstrated that aqueous and methanol extracts of NS seeds exerted a potent sedative and depressive effect on the Central Nervous System (CNS) and analgesic activity *in vivo* (El-Naggar et al. 2003). We considered the possibility that neurotransmitter amino acids could be responsible for these behaviors. After verifying the presence of γ-aminobutyric acid (GABA), glutamate (Glu), aspartate (Asp) and glycine (Gly), and the inhibitory amino acids (IAAs), in the extract quantitatively using high performance liquid chromatography (HPLC), the methanol extract activity on neurotransmitter release was assayed in cultured cortical neurons (El-Naggar et al. 2010). The conclusions of this study suggested an effect of NS methanol extract which modifies the inhibitory amino acids release. This could lead to an increase in the agonist action on their receptors, explaining the sedative and depressive effects observed *in vivo.* Moreover, this effect was coupled with a possible decrease in excitatory transmission that could contribute to the inhibitory response.

Our ongoing interest in the pharmacologic properties of NS prompted us to ascertain the effect of NS methanol extract on brain levels of neurotransmitter amino acids (Asp, Glu, Gly and GABA) in male Wistar rats. For this purpose, we selected five regions, including the thalamus (T), striatum (S), cortex (C), hypothalamus (HT) and hippocampus (HC), and analyzed the effect of NS methanol extract injected intraperitoneally (IP) (2.5 g kg^−1^ body weight) after 1 h and 8 days of treatment. HPLC analysis of amino acids was performed by a previously described method (Márquez et al. 1986) with minor modifications, using equipment and conditions that were previously developed to determine amino acids (Naval et al. 2006). The aim of this study is to evaluate the effect of IP injection of NS methanol extract on changes observed in the aforementioned amino acids and how this may relate to the actions attributed to NS in other research works.

## Materials and methods

### Reagents

Glu, Asp, GABA and Gly were purchased from Sigma (Madrid, Spain). The solvents used for chromatography were acetic acid, triethylamine and methanol of HPLC ultra gradient grade supplied by Tecknochroma (Barcelona, Spain). Dansyl chloride (≥99.0% HPLC for fluorescence BioChemika) was purchased from Sigma (Madrid, Spain). Acetonitrile (HPLC grade) was purchased from Merck (Darmstad, Germany). Lithium carbonate and ethylamine were obtained from Panreac (Madrid, Spain).

HPLC ultra-pure water generated by a Milli-Q system with a resistance value of 18.2 MΩ-cm (Millipore, MA) was used to prepare the aqueous solutions.

Membrane filters (0.45 μm pore size) from Tecknochroma (Barcelona, Spain) were used for filtration in the mobile phase and the samples.

### *Nigella sativa *extract

NS seeds were supplied by the Medicinal and Aromatic Plants Research Institute of Egypt (Cairo, Egypt). Herbarium samples were authenticated by José María Pizarro Domínguez (Herbario MAF, Dep. Biología Vegetal II) and a specimen was placed on deposit in the Herbarium of the Facultad de Farmacia, Universidad Complutense de Madrid, with voucher number MAF 161043.

The methanol extract of the plant was prepared in accordance with the CYTED (Science and Technology Program for Development) protocol for plant species from countries that form part of this Program (CYTED 1993). The seeds were powdered and then extracted in a Soxhlet extractor with hexane. Then the seeds was treated with methanol and kept for further study. Methanol was evaporated in a rotary evaporator under vacuum conditions. A dry blackish-brown extract was obtained and kept at 4 °C until use.

### Animals

Male 8-week-old Wistar rats weighing from 175 to 200 g were used. The animals were housed in groups of five per plastic cage and kept in an acclimatized room for at least 15 days with sawdust as bedding with a 12 h light/dark cycle (lights on at 8:00 and lights off at 22:00). The temperature and humidity in the breeding and exposure rooms were maintained at 23 ± 1 **°**C and 55 ± 5%, respectively. Food and water were accessible *ad libitum*.

The animals were treated in accordance with the current law (an Order enacted on 10 October 1997 and implemented in Law 32/2007 of 7 November 2007). The experiments were performed according to the Guidelines of the Council of the European Union (86/609/EU).

### Experimental design and chemicals

After acclimatization, the animals were grouped so that mean body weight did not differ among the groups. Four groups were created with five rats each, for their evaluation. Two of them, groups 1 and 2 (controls) were IP treated with saline solution. Groups 3 and 4 were IP treated with NS methanol extract at a dose of 0.5 mL saline suspension, amounting to the equivalent of 2.5 g kg^−1^ body weight. One hour after NS injection, the animals in group 1 and group 3 were sacrificed by decapitation. After this, each animal’s brain was dissected and placed on ice prior to removing the five aforementioned brain structures (T, S, C, HT and HC). Group 2 of the animals, designed as a control for the NS treatment lasting 8 days, was injected IP with saline solution daily during this time. Simultaneously, group 4 was injected IP with NS methanol extract at the indicated dose. At the end of this period, both groups of rats were sacrificed and the brain regions extracted as in groups 1 and 3.

### Sample preparation

Tissue samples were weighed and then homogenized by sonication in an ice-cold phosphate buffer solution 50 mM pH 7.5 (1 mg of biological sample in 80 μL of buffer). After this, the homogenates were centrifuged at 18.000 *g* for 5 min at 4 °C and the supernatants were removed, lyophilized and stored at −80 °C for their later dansylation, then measuring the amino acids therein.

### Preparation of amino acid standards

Individual standard stock solutions (1 mM) of amino acids (Asp, Glu, Gly and GABA) were prepared. These stock solutions were then used to prepare a working amino acid mixture solution that contained 5, 10, 15, 20 and 30 μM of each, dissolved in solvent B of gradient. Instances of determining different points on the curve were performed in duplicate. The linearity of the calibration curves was very good (*r* = 0.99) for the four amino acids indicated.

### Dansylation reaction of amino acids

The lyophilized samples of brain tissue homogenates were reconstituted in 0.1 mL of a 40 mM lithium carbonate buffer (pH 9.5), mixed with 50 μL of dansyl chloride 10 mM in acetonitrile and after stirring, the mixture was incubated for 1 h in darkness at room temperature. After that, the addition of 10 μL of ethylamine 2% stopped the reaction. The samples were transferred to an Eppendorf tube and centrifuged for 5 min (13 000 rpm) at 4 °C. The supernatants were filtered through a Hamilton syringe provided with a 13 mm diameter disposable syringe filter (pore size 0.4 μm) and collected to determine HPLC amino acid. The amino acid standards were derivatized in a similar manner.

### Instrumentation and chromatographic conditions for neurotransmitter amino acid analysis

The chromatographic analysis was carried out with the equipment and conditions previously developed to determine the amino acids (Márquez et al. 1986; Naval et al. 2006). An HPLC system Spectra Physics Model SP 8800 Ternary pump (San José, CA) and a photodiode array detector Shimadzu SPD-10 A (Izasa, Madrid, Spain). The data were analyzed using a Spectra Physics Model SP 4400 integrator and software Labnet, Chromdat, Spectra Physics.

Thus, 20 μL of derivatizated sample were injected into the HPLC system using a Rheodyne injection valve. A Waters ODS Spherisorb 150 mm × 4.6 mm I.D. 5 μm packed column (Teknokroma, Barcelona, Spain) was used as a stationary phase, preceded by a guard-column of Spherisorb RP-18, 5 μm, 4 mm ×4 mm. The column flow was 1 mL/min and the stability of the column temperature was ±1 °C/h with respect to 25 °C. The mobile phase was pure methanol (mobile phase A) and 0.6% acetic acid in water/0.008% triethylamine (mobile phase B). The amino acid concentration in the solution was determined by reversed-phase high-performance liquid chromatography and UV detection at 254 nm. Peaks were integrated by the use of a Spectraphysic integrator and they were quantified by comparison with simultaneously prepared amino acid standards.

The concentrations of the amino acids studied in the five brain areas were expressed as pmol/mg of fresh tissue analyzed.

We had previously validated the conditions of chromatographic analysis by HPLC for the valuation of amino acids. We proved that the values obtained in the determination achieved the analytical parameters (linearity, precision, limit of detection and accuracy) that widely confirmed the quality demanded for such methods (Naval et al. 2006). The levels of the four neurotransmitter amino acids were determined in the five brain regions cited in the range of 0.100–1.800 pmol/mg of tissue.

### Statistical evaluation

Free amino acid contents were expressed as pmol/mg tissue. Data, presented as means ± SEM for each group, were calculated from three separate calculations obtained from different brain areas in each rat. The assays of the same sample usually varied by around 2% and the different samples from the same area in different animals varied by 4–7% (less variation when the amino acid displays higher levels). Those variations in the cerebral amino acid levels were attributed to differences among individual animals and were not a result of the assay method.

A Student’s *t*-test was used to compare data statistically. Differences between groups at *p* < 0.05 were considered significant.

## Results

### Determining neurotransmitter amino acids

The HPLC method was applied to determine neurotransmitter amino acids quantitatively in the different rat brain regions analyzed in accordance with the methods described in Materials and Methods. The results are expressed as pmol/mg of the different amino acids analyzed (Asp, Glu, Gly and GABA).

### Effect of NS administration on thalamus region analyzed

**Figure 1.**
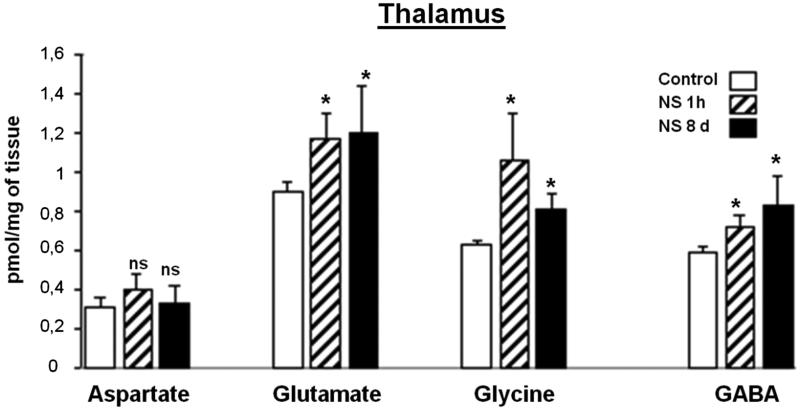
Effect of *Nigella sativa* methanolic extract on neurotransmitter amino acid levels in the rat thalamus after 1** **h and 8** **days of treatment. Data in pmol/mg of tissue are presented as mean** **±** **SEM of five animals per group assayed in triplicate. Statistical significances are given regarding to the corresponding control: ns** **=** **no significant and *p** **<** **0.05.

NS administration produced different alterations in amino acids levels at the two periods of time tested (Figure 1). The results for different amino acids in the T region showed that Glu is a major neurotransmitter, followed by Gly and GABA, respectively. The NS treatment produced a statistically significant increase in these amino acids and moreover, in the case of GABA, this increase was produced in a dose-dependent manner with respect to the number of days of NS administration. However, in the two periods tested, Asp did not show significant changes with respect to the control animals.

### Effect of NS administration on striatum region analyzed

**Figure 2.**
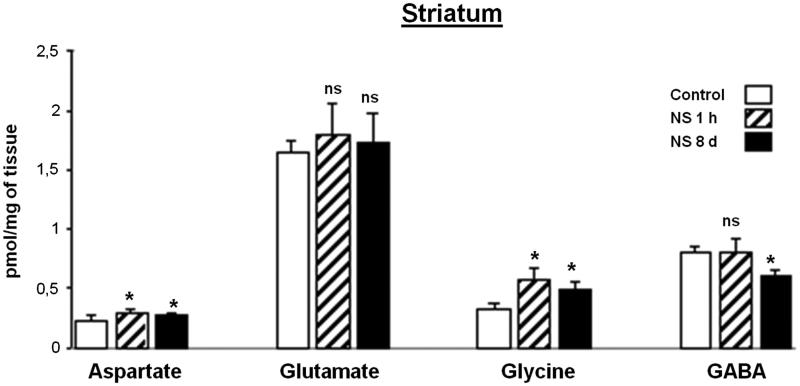
Effect of *Nigella sativa* methanolic extract on neurotransmitter amino acid levels in the rat striatum after 1** **h and 8** **days of treatment. Data in pmol/mg of tissue are presented as mean** **±** **SEM of five animals per group assayed in triplicate. Statistical significances are given regarding to the corresponding control: ns** **=** **no significant and *p** **<** **0.05.

Figure 2 shows the levels of Asp, Glu, Gly and GABA in the S region, 1 h after the NS IP injection and after 8 days of daily treatment. Levels of Asp and Gly were significantly higher than those of the control and decreased simultaneously after 8 days, though they remained above the control value. The level of GABA did not manifest variations after 1 h of treatment with respect to control, but it was significantly lower when the animals were exposed to a longer treatment. Glu did not display significant variations in any of the two periods of NS treatment considered.

### Effect of NS administration on cortex region analyzed

**Figure 3.**
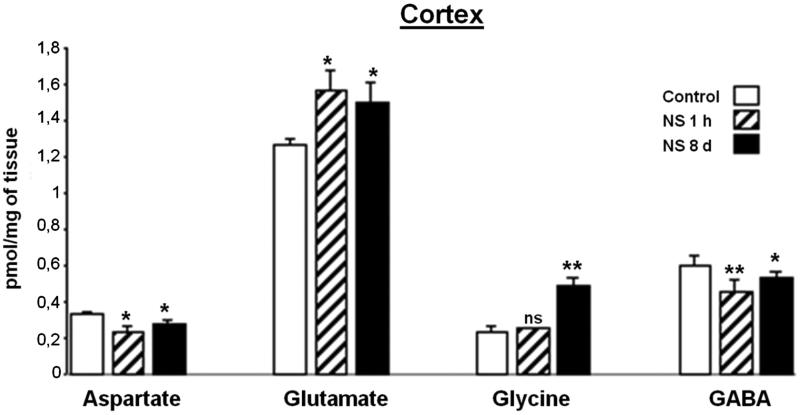
Effect of *Nigella sativa* methanolic extract on neurotransmitter amino acid levels in the rat cortex after 1** **h and 8** **days of treatment. Data in pmol/mg of tissue are presented as mean** **±** **SEM of five animals per group assayed in triplicate. Statistical significances are given regarding to the corresponding control: ns** **=** **no significant, *p** **<** **0.05 and **p** **<** **0.01.

The presence of four neurotransmitter amino acids was quantified in C (Figure 3). The results showed that Glu is the amino acid that is present in this brain region at the greatest quantity. The cortex content of Asp and GABA was significantly reduced by NS treatment and, although their values were lower than the control, it showed a tendency towards returning to control values after 8 days of treatment. Gly underwent a statistically significant increase when the rats were treated during prolonged NS treatment.

### Effect of NS administration on the hypothalamus region analyzed

**Figure 4.**
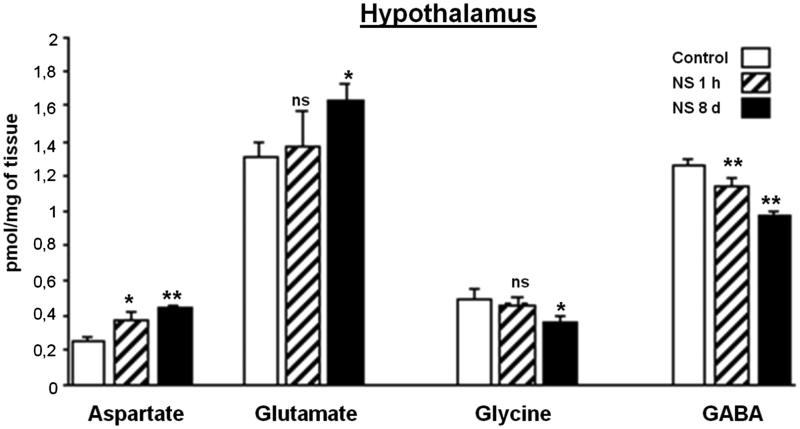
Effect of *Nigella sativa* methanolic extract on neurotransmitter amino acid levels in the rat hypothalamus after 1** **h and 8** **days of treatment. Data in pmol/mg of tissue are presented as mean** **±** **SEM of five animals per group assayed in triplicate. Statistical significances are given regarding to the corresponding control: ns** **=** **no significant, *p** **<** **0.05 and **p** **<** **0.01.

Figure 4 shows the results of the changes of amino acids in HT after NS treatment. The excitatory amino acids (EAAs) Asp and Glu both showed a statistically significant increase in value after eight days of IP injections of NS extract, but Asp showed this behavior from the beginning of the treatment. The inhibitory amino acids, Gly and GABA, underwent a significant decrease in their contents and were especially important for GABA as of the first dose.

### Effect of NS administration on the hyppocampus region analyzed

**Figure 5.**
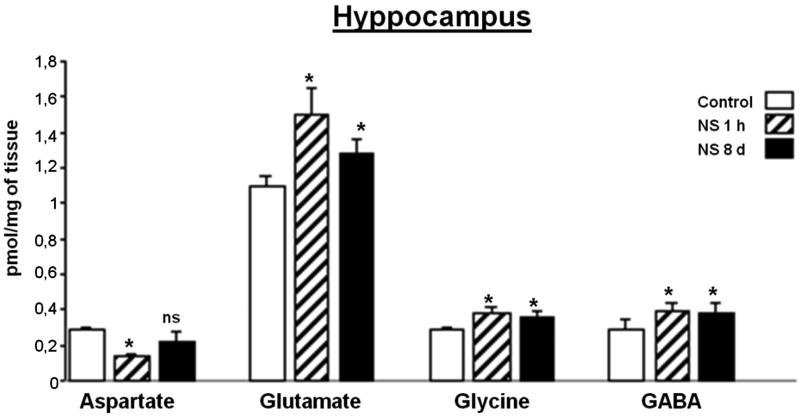
Effect of *Nigella sativa* methanolic extract on neurotransmitter amino acid levels in the rat hyppocampus after 1** **h and 8** **days of treatment. Data in pmol/mg of tissue are presented as mean** **±** **SEM of five animals per group assayed in triplicate. Statistical significances are given regarding to the corresponding control: ns** **=** **no significant and *p** **<** **0.05.

As Figure 5 shows, Glu, Gly and GABA were significantly higher than the control in this region during the two periods of time studied. In comparison, Asp was significantly reduced one hour after first NS dose, but 8 days after NS treatment it showed a tendency towards returning to control values, without reaching the control value.

### Relative percentage variation in neurotransmitter amino acids

Variations in the four amino acids studied, expressed as a relative percentage for the different brain regions analyzed, compared with their control values, are shown in Table 1. The direction of the arrow indicates whether the percentage corresponds to an increase or decrease in the value calculated after the treatment when compared with the control.

**Table 1.** Relative percentage of variation with regard to his value control for Asp, Glu, Gly and GABA in different brain regions using quantitative analysis.

	Thalamus		Striatum		Cortex		Hypothalamus		Hippocampus	
	1 h	8 d	1 h	8 d	1 h	8 d	1 h	8 d	1 h	8 d
Aspartate	28 ↑*	7 ↑*	26 ↑	17 ↑	29 ↓*	16 ↓*	50 ↑*	76 ↑**	53 ↓*	24 ↓
Glutamate	30 ↑*	33 ↑*	9 ↑	5 ↑	24 ↑*	19 ↑*	4 ↑	26 ↑*	36 ↑*	16 ↑*
Glycine	66 ↑*	29 ↑*	75 ↑*	48 ↑*	6 ↑	109 ↑**	7 ↓	28 ↓*	32 ↑*	25 ↑*
GABA	22 ↑*	40 ↑*	1 ↓	24 ↓*	24 ↓**	11 ↓*	10 ↓**	23 ↓**	38 ↑*	32 ↑*

The direction of the arrow indicates if the percentage corresponds with an increase or decrease of the value obtained after the treatment with respect to the control. Statistical significances are given regarding to the corresponding control:

* *p*** **<** **0.05 and

** *p*** **<** **0.01. The percentage without asterisk indicates that the value does not present statistical significance.

The two inhibitory amino acids underwent a decrease in percentage compared to the control at HT. GABA underwent a statistically significant decrease (10%) in this region 1 h after IP injection of NS extract, and the decrease was greater when the treatment was prolonged for 8 days (23%). In C and S, GABA diminished by a statistically significant amount (11% and 24%, respectively) over the same period of time (8 days). The greatest statistical significance increase observed in all of the regions studied took place in an inhibitory amino acid, Gly in the cortex, which increased by 109% compared with the control after 8 days of treatment. Moreover, Gly in T and S displayed the highest percentage variation after the first hour of NS extract injection, of the four amino acids studied.

In terms of the excitatory amino acids, although Glu was the most abundant amino acid in the five brain regions analyzed (stated as pmol/mg tissue), its percentage variation compared with the control was increased moderately by the effect of NS extract. Asp was the only one to undergo a statistically significant increase in T and HT over both treatment times. The values obtained in HT were the highest with statistical significance found for both excitatory amino acids.

## Discussion

As indicated in the Introduction, our primary results demonstrated possible NS action on CNS *in vivo* (El-Naggar et al. 2003), which was confirmed in a later study on cultured cortical neurons (El-Naggar et al. 2010).

We had previously validated the conditions of chromatographic analysis by HPLC for the valuation of amino acids. We proved that the values calculated were in line with analytical parameters (linearity, precision, limit of detection and accuracy) that widely confirm the quality required of such methods (Naval et al. 2006).

The results of this work indicate that, in the untreated animals (groups 1 and 2), Glu was the most abundant excitatory amino acid in the five selected brain structures, whereas the other excitatory amino acid, Asp, had approximately the same quantitative value in these regions. The inhibitory neurotransmitter amino acids Gly and GABA displayed similar values in both HC and T. Only in HT did the control levels of Glu and GABA have a similar quantity, the latter reaching a higher value than the levels detected in other regions analyzed.

Our results seem to agree with the major presence of Glu observed in different regions of the rat brain, and with the results obtained for Asp by other researchers (Balcom et al. 1976; Noor et al. 2012). These authors compared classical methods to a microwave fixation technique that avoids possible changes in *postmortem* amino acid levels, having found no differences between these procedures.

As for the high level of GABA we detected in the HT, recent publications by other authors also point out that there is a major presence of this amino acid in this region of the *Suncus murinus* brain compared with its content in all other regions (Chan et al. 2010). The same authors have described a similar quantitative value for Glu and GABA in this region, in different species of animals analyzed, which is in line with our results.

The evaluation of NS effects on Asp, Glu, Gly and GABA levels has shown that its administration leads to significant changes in the content of the four neuroactive amino acids in the rat brain. These effects seem to manifest very quickly, most often 1 h after administration of the NS extract. In addition to expressing the amino acid content in pmol/mg of tissue, we have individually analyzed its percentage variation compared with the control after treatment in each of the regions studied. The objective was to gather all of these variations on one single table and make discussion of the results easier. We only considered the results with a statistical significance of up to 0.05 to be of importance.

In the hypothalamus, 1 h after administration of one dose of NS extract, the Asp level increased (50%) and GABA decreased (10%) significantly. When the treatment was prolonged to 8 days, the four amino acids continued to behave as observed over the short time period but now showing statistical significance. Over this period of time and at the dose used in this study, NS produces an increase in EAAs, principally Asp, and a similar decrease in IAAs, Gly and GABA. Excitatory amino acids were detected at significant concentrations in presynaptic boutons of a variety of important hypothalamic nuclei, and different EAA receptor subtypes were found in different areas of this region (Brann & Mahesh 1994). The effect of NS treatment could produce an EAAs hypothalamus increase, acting at the level of neuroendocrine regulation of a variety of hormonal systems, with the largest amount of data available on the control of gonadotropin hormone-releasing hormone (GnRH) release. In a subsequent study regarding the effect of NS oil on hypercholesterolemic rats, the results revealed a significant increase in the male fertility index. This increase was attributed to the antioxidant and hypolipidemic effects of administering NS (Samir Bashandy 2007). With our results, it is possible to add more information to their conclusions. The effect of increasing levels of EAAs would influence the secretion of GnRH, which would have repercussions on the male reproductive system or sexual hormones. At the present time, the plant is being used commercially as a uterine contraction stimulant and as a natural remedy for amenorrhea and dysmenorrhea (El-Dakhakhny 1965; Goreja 2003). Another study (Keshri et al. 1995) showed that the active hexane extract in NS displayed significant antifertility activity in rats, and it has been found to be a contraceptive with uterotrophic activity (Saleem et al. 2012). All these effects would be explained by the increase in EAAs and the decrease in IAAs observed in HT in our results. This decrease would favour equilibrium between both types of neurotransmitters, in favour of the first one.

The hippocampus was the structure in which all of the amino acids underwent significant change as of the very first dose of NS extract. Glu, Gly and GABA maintained their increases (16, 25 and 32%, respectively) after 8** **days of treatment. The decrease observed in Asp after the animals received the first dose lost its statistical significance after 8** **days of treatment. Most of the excitatory innervation in the HC, including pyramidal cells, is glutamatergic and the function of these neurons is modulated by inhibitory GABA-releasing interneurons (Frotscher et al. 1984; Freund & Burzaki 1988). These neurons are known to mediate recurrent inhibition of feed forward inhibition to the pyramidal neurons (Alger & Nicoll 1982). It has been shown that the massive amounts of GABA released simultaneously with the EAAs in the HC may constitute an important protective mechanism against the excessive release of the latter, counteracting the harmful effects which lead to neuronal death. The release of IAAs may limit excitation and prevent reaching neurotoxic levels (Oja et al. 1990; Sivilotti & Nistri 1991; Watanabe et al. 2002). The antiepileptic effects of the main constituents of NS seed have been evaluated using different agents**-**induced seizures (Hosseinzadeh & Parvardeh 2004; Raza et al. 2006, 2008; Ezz et al. 2011), and the involvement of GABA receptors in this process has been acknowledged, as well. Our results showed that GABA and Gly remained at similar high levels throughout the treatment, remaining higher at the end of the treatment than did Glu. These IAAs could be preferably controlling the excitation of pyramidal cells. Likewise, these results could explain the effects we observed in mice when aqueous or methanol extract was administered to the animals. Our previously published results had indicated that, when they were treated with these extracts, a significant reduction occurred in spontaneous motility, with decreased exploratory conduct and concomitant decreased motor coordination induced (El-Naggar et al. 2003).

Another of the interesting conclusions reached by this study involves Gly. The levels of this inhibitory amino acid increased in all of the cerebral regions analyzed, with the exception of HT, when the rats were injected with NS extract. This inhibitory amino acid increased significantly compared with the control, after administration of the first dose, with the exception of the cortex. After 8** **days of treatment, these levels remained higher than the control values and became especially high in the case of cortex (109%). The increase in Gly has been related with anti-inflammatory, cytoprotective and immunomodulator action (Pereira et al. 2009; Weinberg et al. 2016). The hyperpolarization produced in the cell by the activation of the glycine receptor and the subsequent blockage of calcium entry may be responsible for suppressing the formation of free radicals and inflammatory cytokines (Zhong et al. 2003). The increase in Gly observed in our results could be related with the potent immunomodulatory and immunotherapeutic potentials observed in NS seed and its constituents (Al-Ghamdi 2001; El-Dakhakhny et al. 2002; Salem 2005), as well as with the antioxidant and anti-inflammatory action observed in the methanol extract obtained not only from the seeds but from another parts of the plant (Bourgou et al. 2012).

GABA had an increased value in T and HC, whilst in S, HT and C it decreased during the two time periods studied. A decrease in the levels of this neurotransmitter in these cerebral regions can be explained by a reduction of glutamate levels, because this amino acid acts as a precursor to GABA synthesis. Although our results showed that Glu increased in these three regions, its percentage compared with the control is lower than that found in the others.

## Conclusions

This study demonstrates the effects of a methanol extract of NS on amino acid levels in rat brains. In conclusion, these results are consistent with our previous hypothesis, in which we suggested that NS methanol extract could alter the release of endogenous amino acids in the CNS, changing the levels of inhibitory and excitatory amino acids in different brain regions. These results seem to explain some of the effects attributed to this plant in different scientific studies and constitute an advancement towards greater knowledge about this seed and its possible pharmacological action. This study is of interest when considering that some neurodegenerative diseases are related to an imbalance in amino acid levels in the CNS and, from this perspective, NS could be considered a potential aid in the treatment of some neurological disorders.
